# Overview of Performance Indicators of Prostate Cancer Screening Trials

**DOI:** 10.1016/j.euros.2025.08.004

**Published:** 2025-09-03

**Authors:** Meike J. van Harten, P.M. Willemse, R.C.N. van den Bergh, M.J. Roobol

**Affiliations:** a Erasmus University Medical Centre, Cancer Institute, Rotterdam, The Netherlands; b Department of Urology, Cancer Center, University Medical Center Utrecht, Utrecht, The Netherlands

**Keywords:** Prostate cancer, Screening, Trials, Performance indicators

## Abstract

**Background and objective:**

Prostate cancer (PCa) screening is highly debated in urological literature and in the society. In 2021, the EU Commission proposed pilot studies to explore the feasibility of risk-based population-based PCa screening. The PRostate cancer Awareness and Initiative for Screening in the European Union (PRAISE-U) project aims to evaluate the feasibility and effectiveness of screening pilots by applying specific performance indicators (PIs). A comparison of the PIs of new screening initiatives with those of previous trials can provide valuable insights. This study offers an overview of the data from the largest ongoing and past PCa screening trials, translating these into uniformly calculated PIs to serve as a reference for future studies.

**Methods:**

A narrative review was conducted. Population-based screening trials with ≥1000 participants were included. The following main indicators were extracted and presented: participation rate, proportion of screening positives, applied risk stratification tools, proportion of magnetic resonance imaging (MRI) scans and MRI compliance, proportion of biopsies and biopsy compliance, cancer detection rates, and proportion of clinically significant PCa cases.

**Key findings and limitations:**

Ten trials were included for analysis (European Randomised Study of Screening for Prostate Cancer; Cluster randomised trial of prostate-specific antigen (PSA) testing for Prostate cancer; Prostate, Lung, Colorectal and Ovarian Cancer Screening Trial; Prostate Cancer Early Detection Study Based on a “Baseline” PSA value in Young Men; Göteborg-2; Early Prostate Cancer Detection Programme; Organised Prostate cancer Testing; STHLM3-MRI; ProScreen; and BARCODE1). Participation rates varied widely (from 12% to 89%), affected largely by study start year (associated with opportunistic PSA testing), study design, and age of participants. PSA positivity rates ranged from 0.8% to 29%, associated mainly with age, usage of repeat PSA tests, and socioeconomic factors. The proportion of MRI scans among participants ranged from 0.6% to 11%, dependent mainly on indication, number of positive multivariable algorithms, and age. Biopsy rates among participants ranged from 0.5% to 25%, affected by risk stratification strategies, compliance, and biopsy trigger. The detection rate of clinically significant PCa, calculated as the proportion of any PCa cases, ranged from 41% to 82%.

**Conclusions and clinical implications:**

The PIs presented here may serve as a reference for other ongoing national screening pilot studies such as PRAISE-U. Differences in time period, population, informed consent strategy, and diagnostic algorithm lead to a wide range of PIs. These factors must be considered and taken into account to correctly compare the PIs across different national screening pilots.

**Patient summary:**

The effectiveness of a screening program can be assessed using performance indicators. In this article, we present a comprehensive overview of the performance indicators for the most well-known prostate cancer (PCa) screening studies. This can serve as a reference for ongoing and future PCa screening initiatives. However, it is important to consider that several factors, such as differences in target population, protocols, and diagnostic algorithms may affect outcomes when comparing these studies.

## Take Home Message

Performance indicators enable a comparison of prostate cancer screening data across trials, but various factors influence their validity. Differences in populations, invitation methods, screening protocols, and technologies must be considered when interpreting results.

## 1 Introduction

In the early 1980s, it became clear that prostate-specific antigen (PSA) could also serve as a marker for the presence of prostate cancer (PCa), leading to its utilization as a screening tool [1,2]. Following this discovery, several studies started investigating the effectiveness of PCa screening, with the Prostate, Lung, Colorectal and Ovarian (PLCO) Cancer Screening Trial and the European Randomised Study of Screening for Prostate Cancer (ERSPC) being the first population-based PCa screening randomized trials, both starting in 1993 [3,4]. According to the Wilson and Jungner [5] criteria, screening in general is a systematic process of identifying nonsymptomatic individuals with a disease at an early, treatable stage using an acceptable and cost-effective test, applied to a population where the condition is a significant health concern and the benefits of early detection outweigh the harms. In addition, the World Health Organization recommends that at least 70% of the target population should participate in screening to ensure the effectiveness of a population-based screening program [6].

Despite the current general agreement that PSA screening reduces the number of cases with metastatic disease and those dying from the disease, implementation of population-based PCa screening remains limited. The main reason for this is the coinciding overdiagnosis and resulting overtreatment when applying a PSA-based algorithm. The balance between advantages and side effects of this PSA-based algorithm in combination with a random systematic prostate biopsy has therefore been generally considered as unfavorable [7]. In recent decades, novel diagnostic tests have emerged. Usage of urine and biomarkers, magnetic resonance imaging (MRI), and multivariable risk calculators has been integrated gradually in the diagnostic pathway, allowing for a more individual and sensitive approach and thereby affecting the harm-benefit ratio of PCa screening positively.

Within the setting of the “Europe’s beating cancer” plan, in 2021, the European Union updated their cancer screening recommendation to also include PCa. This opened the way to explore the feasibility of organized, population-based PCa screening in the different member states within the European Union [8]. Following this recommendation, the EU4Health program provided funding to support the startup of national PCa screening pilot studies. A scientific consortium working on realizing the initiation of such pilot studies is the PRostate cancer Awareness and Initiative for Screening in the European Union (PRAISE-U) [9]. In short, five pilots started implementing a population-based PCa screening program following an algorithm that, next to PSA testing in men aged 50–69 yr, incorporates a double risk stratification step (before and after MRI). However, each pilot adopted different implementation approaches to these risk stratification steps tailored to their regional health care systems to enhance feasibility and effectiveness.

The effectiveness of a screening program should be assessed by well-defined performance indicators (PIs). For colorectal and breast cancer screening, European guidelines exist regarding the collection of these PIs [10,11]. Recently, in the context of PRAISE-U, these PIs have been developed for use in PRAISE-U and other national initiatives on PCa screening [12]. Any evaluation of performance is based on having a benchmark. The aim of this manuscript is to provide an overview of PIs from the most well-known ongoing and past PCa screening trials.

## 2 Methods

The data presented are based on a narrative review including the larger studies on population-based PCa screening since 1993, a year marked by the start of two landmark studies on population-based PCa screening [13,14]. The exclusion criteria were trials and pilots not using PSA as the primary screening tool and studies with <1000 participants, to ensure that the included studies were representative of the general population. As a result, the MVP study was excluded due to its sample size (*n* < 1000) [15] and the ReIMAGINE study was excluded because results are not yet available [16].

The studies identified were divided into two groups based on screening diagnostics: (1) studies using only PSA as the main indication for prostate biopsy and (2) studies using PSA followed by individual (multivariable) risk stratification (biomarker/risk calculator/MRI) as an indication for prostate biopsy.

A description of the screening pathway is presented for each study, including information on the total number of men invited, actual participants, screening tools, screening interval applied, available follow-up, and relevant study outcomes based on the identified PIs for PCa screening.

### 2.1 Performance indicators

The most accessible and commonly available PIs, developed within PRAISE-U, were selected to facilitate a comparison between the trials identified through the PubMed search and by applying our exclusion criteria (Table 1) [12]. Depending on availability, we also aimed to include cancers detected after screening and those detected outside the screening program.

**Table 1:** Description of performance indicators used for analyses

Performance indicator	Definition
Participation rate	Proportion of men who had their PSA tested after invitation
Proportion of PSA positives	Total number of men with an elevated PSA level divided by the total number of men participating
Proportion of MRI scans	The total number of men who received an MRI scan divided by the total number of men participating
MRI compliance	Proportion of men indicated for MRI who actually received an MRI scan
Proportion of biopsy	The total number of men undergoing biopsy divided by the total number of men participating
Biopsy compliance	Proportion of men indicated for a biopsy who actually received biopsies
Detection rate	Total number of detected PCa cases among all men invited (%), participants (%), PSA positives (%), MRI scans (%), and biopsies (%)
Detection of csPCa	Proportion of PCa cancers found (%) with a Gleason score of ≥7 or ISUP ≥2

csPCa = clinically significant PCa; ISUP = International Society of Urological Pathology; MRI = magnetic resonance imaging; PCa = prostate cancer; PSA = prostate-specific antigen.

## 3 Results

**Fig. 1:**
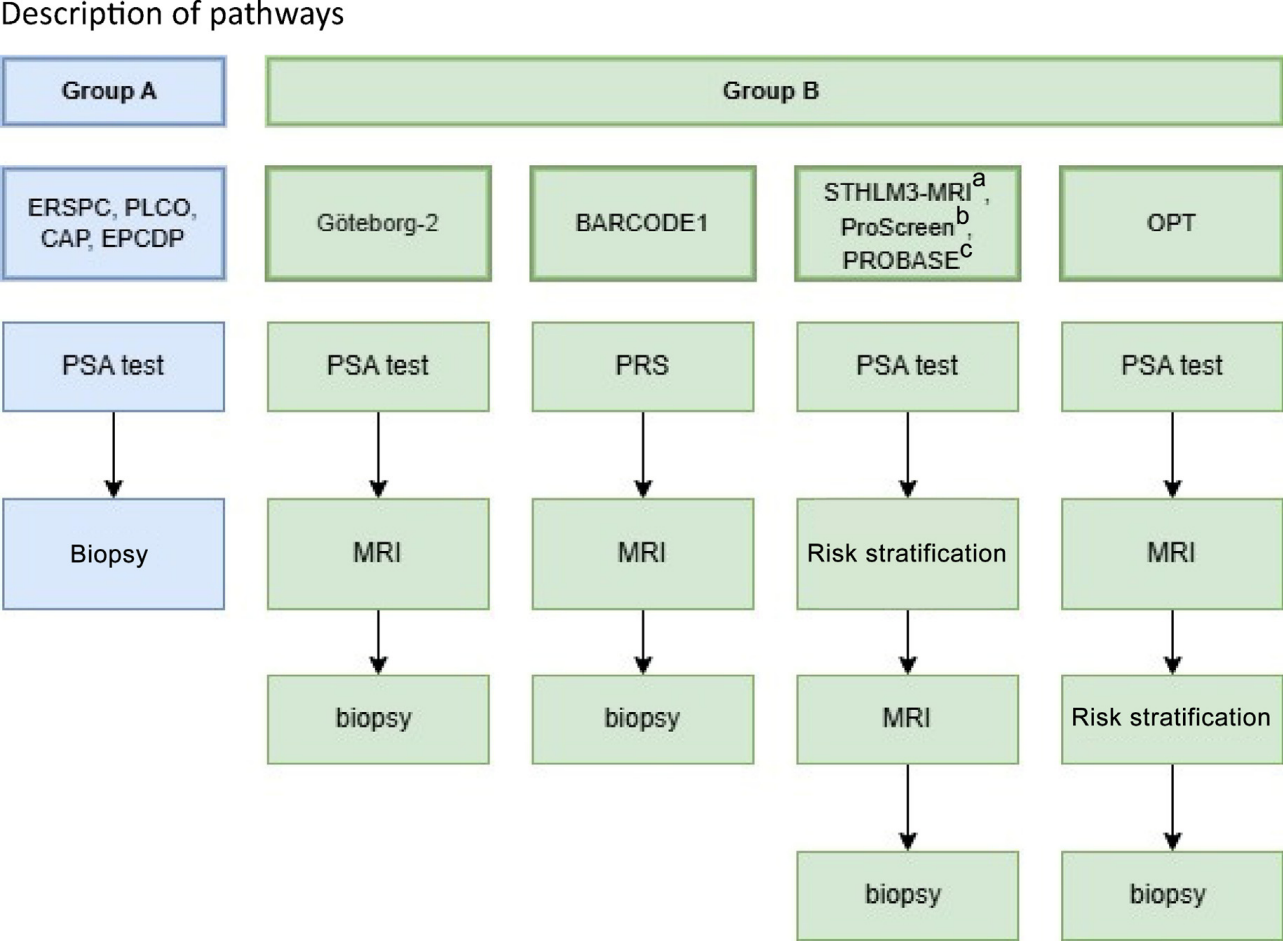
Description of pathways from included studies. ERSPC = European Randomised Study of Screening for Prostate Cancer; PLCO = Prostate, Lung, Colorectal and Ovarian Cancer Screening Trial; CAP = Cluster randomised trial of PSA testing for Prostate cancer; EPCDP = Early Prostate Cancer Detection Programme; PROBASE = Prostate Cancer Early Detection Study Based on a “Baseline” PSA value in Young Men; OPT = Organised Prostate cancer Testing; PSA = prostate-specific antigen, MRI = magnetic resonance imaging.

In total, ten studies were found to be eligible for inclusion. Group A (PSA/biopsy) consists of ERSPC [3], PLCO Cancer Screening Trial [4,17], Cluster randomised trial of PSA testing for Prostate cancer (CAP) [18], and Early Prostate Cancer Detection Programme (EPCDP) [19]. Group B (PSA/risk stratification/biopsy) consists of Göteborg-2 [7,20], Prostate Cancer Early Detection Study Based on a “Baseline” PSA value in Young Men (PROBASE) [21], Organised Prostate cancer Testing (OPT) [22,23], STHLM3-MRI [24,25], ProScreen [26,27], and BARCODE1 [28,29]. Fig. 1 illustrates the different steps involved in the pathways of the included studies.

A detailed description of study details and the abovementioned PIs for each study can be found in Table 2, Table 3, with corresponding references in Table 4.

**Table 2:** Group A from included studies

	ERSPC Europe	PLCO USA	CAP UK	EPCDP Lithuania
Total invited eligible group (*N*)	162 241	76 693	415 357	655 487
Total participation^a^, *N* (%)	NA	NA	NA	459 667 (70)
Diagnostic tools	PSA, SBx	PSA, DRE, biopsy	PSA, SBx	PSA, DRE, biopsy
Screening interval	Every 2 or 4 yr	Annual	Single PSA	2 (>2009)
Follow-up (yr)	16	17	17	10
Study design	RCT	RCT	RCT	National screening program
Study outcome	PCa mortality	PCa mortality	PCa mortality	PCa
Study groups	Intervention	Intervention	Intervention	NA
*N*	72 890	38 340	189 326	655 487
Age (yr)	55–69	55–74	50–69	50–74
Mean	60.9	NA	NA	NA
Median	60.3	NA	59	NA
Method of invitation	Population registry	Recruitment	GP, single invitation	By opportunity at GP
PSA threshold for referral (ng/ml)	≥3	≥4	≥3	≥3
Participation rate in groups^a^, *N* (%)	60 261 (83)	34 175 (89)	64 425 (34)	459 667 (70)
Proportion of PSA test positives^b^, *N* (%)	16 988 (23)	9755 (29)	6855 (11)	128 909 (13)
Proportion of MRI scans^c^, *N* (%)	NA	NA	NA	NA
Compliance^d^ (%)				
Proportion of biopsies^e^, *N* (%)	15 116 (25)	4424 (13)	5848 (9)	46 180 (10)
Compliance^f^ (%)	86	45	85	36
Cancer detection				
Number of cancers found	8444	5574	12 013	26 896
Screen-detected cancers	4957	NA	6554	16 061
Non–screen-detected cancers	3487	NA	5459	10 835
Among all men invited (%)	6.8	15	3.5	2.5
Among participants (%)	8.2	16	10	3.5
Among PSA positives (%)	29	57	96	13
Among MRI scans (%)	NA	NA	NA	NA
Among biopsies (%)	33	NA	NA	35
Gleason grade ≥2^g^, *N* (%)	2157 (44)	2298 (41)	3287 (50)	NA

ERSPC = European Randomised Study of Screening for Prostate Cancer; PLCO = Prostate, Lung, Colorectal and Ovarian Cancer Screening Trial; CAP = Cluster randomised trial of PSA testing for Prostate cancer; EPCDP = Early Prostate Cancer Detection Programme; PROBASE = Prostate Cancer Early Detection Study Based on a “Baseline” PSA value in Young Men; OPT = Organised Prostate cancer Testing; PSA = prostate-specific antigen, MRI = magnetic resonance imaging.

a Participation rate is defined as the proportion of men who had their PSA level tested after invitation.

b Proportion of PSA test positives is defined as the total number of men with an elevated PSA level divided by the total number of men participating.

c Proportion of MRI scans is defined as the total number of men who received an MRI scan divided by the total number of men participating.

d MRI compliance is defined as the proportion of men indicated for MRI (elevated PSA) who actually had an MRI scan.

e Proportion of biopsies is defined as the proportion of men undergoing biopsy divided by the total number of men participating.

f Biopsy compliance is defined as the proportion of men indicated for biopsy (elevated PSA/suspected MRI) who actually had a biopsy.

g The proportion of grade group ≥2 is calculated as part of the total number of any PCa cases found.

**Table 3:** Group B from included studies

	Gothenburg 2 Sweden		PROBASE Germany		OPT Sweden	STHLM3-MRI Sweden		ProScreen Finland	BARCODE1 UK
Total invited eligible group (N)	37 887		>400 000		68 060	49 118		60 745	40 292
Total participation^a^, *N* (%)	17 980 (47)		46 642 (12)		23 855 (35)	12 750 (26)		NA	6393 (16)
Screening tools	PSA, MRI, SBx, TBx		PSA, DRE, mpMRI, TBx, SBx		PSA, PSA-D, bpMRI, SBx, TBx	PSA, STHLM3, bpMRI, SBx, TBx		PSA, Kallikrein risk score, mpMRI, SBx, TBx	PRS, mpMRI, transperineal biopsy, PSA
Screening interval (yr)	2–8		2–5		2–6	NA		2–6	NA
Follow-up in publication (yr)	5 (recruitment)		5.9		1	3		3	Ongoing
Study design	RCT		RCT		Regional screening program	RCT		RCT	Single arm, cohort
Primary study outcome	Clinically insignificant PCa (Gleason 3 + 3)		Distant metastatic PCa at age 60 yr		Participation, PSA distributions, PI-RADS scores, cancer detection and treatment	Clinically significant cancer (Gleason 3 + 4)		Prostate cancer mortality	Clinically significant cancer (Gleason 3 + 4)
**Study groups**	**Experimental**	**Reference**	**Immediate**	**Deferred**	**NA**	**Biomarker**	**MRI enhanced**	**Screening**	**NA**

*N*	11 986	5994	23 341	23 301	68 060	5134	7609	15 201	40 292
Age (yr)	50–60	50–60	45	50	50	50–74	50–74	50–63	50–69
Mean	NA	NA	45.5	45.5	50	NA	NA	57.3	61
Median	56	56	NA	NA	50	61	61	NA	NA
Method of invitation	Letter	NA	E-mail	E-mail	E-mail	Mail	Mail	Mail	Letter
PSA threshold for referral (ng/ml)	≥3	≥3	≥3	≥3	≥3	STHLM3 ≥0.15	≥3	≥3	PRS ≥90th percentile
Participation rate in groups^a^, *N* (%)	NA	NA	23 301 (99.8)	23 194 (99.5)	23 855 (35)	NA	NA	7744 (51)	6393 (15)
Proportion of PSA test positives^b^, *N* (%)	796 (6.6)	405 (6.8)	186 (0.8)	DRE = 6537	696 (2.9)	413 (8)	929 (12)	752 (9.7)	745 (12)
Proportion of MRI scans^c^									
N (%)	762 (6.4)	384 (6.4)	147 (0.6)	NA	645 (2.7)	1	846 (11)	509 (6.6)	551 (8.6)
Compliance^d^ (%)	96	95	79		93		91	97	74
Proportion of biopsies^e^									
*N* (%)	300 (2.5)	348 (5.8)	120 (0.5)		221 (0.9)	326 (6.3)	338 (4.4)	262 (3.4)	468 (7.3)
Compliance^f^ (%)	90	86	65	37	72	79	92	99	63
Cancer detection									
Number of cancers found	176	140	48	2	137	180	233	219	187
Screen-detected cancers								161	
Non–screen-detected cancers								58	
Among all men invited (%)	1.5	2.3	0.2	0.009	0.2	NA	NA	1.1	0.5
Among participants (%)	1.5	2.3	0.2	0.009	0.6	3.5	3.1	2.1	2.9
Among PSA positives (%)	22	36	26	0.03	20	44	25	21	25
Among MRI scans (%)	23	37	33	NA	21	NA	28	32	34
Among biopsy (%)	59	40	40	5.4	62	55	69	62	40
Gleason grade ≥2 (%)	110 (63)	68 (49)	33 (69)	0	93 (68)	119 (66)	192 (82)	128 (80)	103 (55)

ERSPC = European Randomised Study of Screening for Prostate Cancer; PLCO = Prostate, Lung, Colorectal and Ovarian Cancer Screening Trial; CAP = Cluster randomised trial of PSA testing for Prostate cancer; EPCDP = Early Prostate Cancer Detection Programme; PROBASE = Prostate Cancer Early Detection Study Based on a “Baseline” PSA value in Young Men; OPT = Organised Prostate cancer Testing; PSA = prostate-specific antigen, MRI = magnetic resonance imaging.

a Participation rate is defined as the proportion of men who had their PSA level tested after invitation.

b Proportion of PSA test positives is defined as the total number of men with an elevated PSA level divided by the total number of men participating.

c Proportion of MRI scans is defined as the total number of men who received an MRI scan divided by the total number of men participating.

d MRI compliance is defined as the proportion of men indicated for MRI (elevated PSA) who actually had an MRI scan.

e Proportion of biopsies is defined as the proportion of men undergoing a biopsy divided by the total number of men participating.

f Biopsy compliance is defined as the proportion of men indicated for a biopsy (elevated PSA/suspected MRI) who actually had a biopsy.

**Table 4:** Overview of included studies and corresponding references

Study	References
ERSPC	[33]
PLCO	[48,49]
CAP	[18,50]
EPCDP	[19]
Göteborg-2	[7,51]
PROBASE	[36]
OPT	[23]
STHLM3-MRI	[25]
ProScreen	[26]
BARCODE1	[28]

ERSPC = European Randomised Study of Screening for Prostate Cancer; PLCO = Prostate, Lung, Colorectal and Ovarian Cancer Screening Trial; CAP = Cluster randomised trial of PSA testing for Prostate cancer; EPCDP = Early Prostate Cancer Detection Programme; PROBASE = Prostate Cancer Early Detection Study Based on a “Baseline” PSA value in Young Men; OPT = Organised Prostate cancer Testing; PSA = prostate-specific antigen, MRI = magnetic resonance imaging.

### 3.1 Group A

1. ERSPC (ISRCTN49127736)—ERSPC is a multicenter randomized screening study in Europe, starting in 1993, and recruitment of participants was completed in 2003, except for France where it was completed in 2005 [3]. Participants were identified through population registries in eight European countries (the Netherlands, Belgium, Sweden, Finland, Italy, Spain, Switzerland, France). Men aged 50–74 were randomized into a screening or an intervention arm. In the intervention arm, men were offered a PSA test with a screening interval of every 4 years (every 2 years in Sweden). For the intervention arm, if PSA was ≥3 ng/ml, men were referred for systematic biopsy (sextant in first two decades). In France, only two screening rounds were conducted. Belgium, Finland, and Spain each conducted three screening rounds. The Netherlands had five screening rounds, while Swedish men were screened up to ten times.
2. PLCO (NCT00002540)—As part of a larger screening initiative for cancers, the PLCO trial randomly assigned participants aged 55–74 yr between 1993 and 2001 to either an intervention or a screening arm [4,17]. In total, ten screening centers in the USA recruited the participants. In the intervention arm, participants underwent annual PSA testing for 6 yr and received a digital rectal examination (DRE) annually for 4 yr. A PSA level of ≥4 ng/ml was considered elevated, and patients with elevated PSA were therefore referred for a biopsy.
3. CAP (ISRCTN92187251)—The CAP study started in 2001 in the UK [18]. Recruitment was completed in 2009 and follow-up ended in 2016. Primary care practices in England and Wales were randomized to an intervention or a control group. Within the intervention group, general practitioners (GPs) identified men aged 50–69 yr from their registry and sent them a single invitation letter for a single PSA test. A PSA threshold of ≥3 ng/ml was applied, followed by a systematic biopsy for those with an elevated PSA level.
4. EPCDP—In Lithuania, since 2006, all asymptomatic men aged 50–74 yr and those aged 45–49 yr with a positive family history can have their PSA levels tested free of charge by their GP when they visit their GP for any reason [19]. Between 2006 and 2009, screening was offered annually. From 2010 onward, screening is offered biennially. Men are referred for biopsy for PSA ≥3 ng/ml and if the DRE is suspicious.

### 3.2 Group B

1. Göteborg-2 (SRCTN94604465)—Starting in 2015, men aged 50–60 yr living in Göteborg (Sweden) and surrounding municipalities, identified through a population registry, received a physical letter to participate [7,20]. These men were first randomized into a screening or control group at a ratio of 2:1. The control group did not undergo any type of screening. Men in the screening group were subsequently assigned to three different groups with different screening strategies at a ratio of 1:1:1. The strategy of the first group (reference arm) involves men with a PSA level of ≥3 ng/ml undergoing MRI, followed by a systematic biopsy regardless of MRI findings. For the second group (experimental group), PSA threshold and prostate MRI are the same, but only men with a Prostate Imaging Reporting and Data System (PI-RADS) score of 3–5 undergo targeted biopsy. The third group is identical to group 2, but with a PSA threshold of ≥1.8 ng/ml being applied.
2. PROBASE—This study started inviting men aged 45 yr in four different sites in Germany by mail in 2014 [21]. Recruitment of participants was completed in 2019. Invitations were sent out through municipal population registries. Participants were randomized into an immediate screening arm or a delayed screening arm at a ratio of 1:1. In the immediate screening arm, participants will have their PSA tested (ie, at age 45 yr). A second PSA test was performed 2 wk later in case PSA ≥3 ng/ml. This result was used to classify the participants into low-risk (PSA <1.5 ng/ml), intermediate-risk (PSA 1.5–2.99 ng/ml), or high-risk (PSA ≥3 ng/ml) group. Men with a low risk are recommended to undergo rescreening in 5 yr. Those with a PSA level of 1.5–2.99 ng/ml are advised to undergo rescreening in 2 yr. For a high risk, men are referred to multiparametric MRI (mpMRI) and a targeted and/or systematic biopsy. In the delayed arm, participants will have their PSA tested at the age of 50 yr. However, a DRE is performed annually until the age of 50 in the delayed arm.
3. OPT—Men aged 50 yr received invitation letters in two Swedish regions [22,23]. These automatically produced invitations are sent by regular mail. Men with a PSA level of ≥3 ng/ml were referred for biparametric MRI. Next, risk stratification took place based on PSA, PSA density (PSA-D), and PI-RADS results, with men being classified into low- and high-risk groups. Participants were defined as those having a low risk if they had PI-RADS 1–3 and PSA-D <0.15 ng/mL^2^. The high-risk group comprised participants with a PSA-D of ≥0.15 ng/mL^2^ or PI-RADS 4–5. The high-risk group was referred for a systematic and/or targeted biopsy (biopsy protocol depended on the region).
4. STHLM3-MRI (NCT03377881)—Men aged 50–74 yr, living in Stockholm, Sweden, were randomly selected from the Swedish Population Registry and received an invitation by mail to participate [24,25]. Recruitment took place from 2018 to 2020. Men with a PSA level of <1.5 ng/ml were considered to be at a low risk and were recommended to have their PSA retested in 6 yr. The STHLM3 risk score is a PCa screening tool that combines information on age, total PSA, free PSA, human-kallikrein 2, previous biopsy results, family history of PCa, and genetic markers to assess an individual's risk of clinically significant PCa (csPCa; Gleason score ≥3 + 4). The STHLM3 test was subsequently performed for participants with a PSA level of ≥1.5 ng/ml. Men with a PSA level of ≥3 ng/ml or STHLM3 ≥0.11 were considered to be at an elevated risk and were randomized into the biomarker or MRI-enhanced group, at a ratio of 2:3. The biomarker group received systematic prostate biopsies. The MRI-enhanced group was first referred for biparametric MRI. If the MRI turned out positive (PI-RADS ≥3), targeted and systematic biopsies were performed.
Men with PSA 1.5–3 ng/ml and STHLM3 <0.11 were considered to have a low risk and were advised to repeat PSA screening in 2 yr.
5. ProScreen (NCT03423303)—All men aged 50–63 yr who were living in Helsinki and Tampere, Finland, were selected from a population registry [26,27]. These men were randomized into a screening or control group at a ratio of 1:3. Within the screening group, men received a mail with an invitation to participate and additional questionnaires about prior PSA measurements and biopsies, among others. If PSA was elevated (≥3 ng/ml), the 4-kallikrein risk score (total PSA, free PSA, intact PSA, and human-kallikrein-2) was determined. Age and the results of the 4-kallikrein markers were consequently used to calculate the risk score of high-grade PCa, ranging from 0% to 100%. A cutoff of ≥7.5% is being applied to identify men at risk and refer them to the urology department. All men at risk undergo mpMRI. Those with a PI-RADS score of 3–5 are advised to undergo a targeted biopsy. Those with a negative MRI result but a PSA-D of ≥0.15 ng/mL^2^ are referred for systematic biopsies. Rescreening was based on initial PSA levels: men with PSA ≥3.0 ng/ml were reinvited after 2 yr, men with PSA 1.5–2.99 ng/ml were reinvited after 4 yr, and men with PSA <1.5 ng/ml were advised to undergo rescreening after 6 yr.
6. BARCODE1 (NCT03857477)—Men aged 55–69 yr were recruited from 69 primary care centers across the UK. They were invited by letters to provide saliva samples, from which germline DNA was analyzed to calculate a polygenic risk score (PRS) based on 130 genetic variants. Men with a PRS of ≥90th percentile were considered to be at a high risk and were referred for mpMRI and transperineal biopsy, regardless of their PSA levels. All biopsies were performed systematically, with additional targeted biopsies conducted for any lesions identified on MRI. Participants with negative biopsies were followed up with screening annually for 5 yr.

### 3.3 Description of PIs and interpretation

#### 3.3.1 Participation

**Fig. 2:**
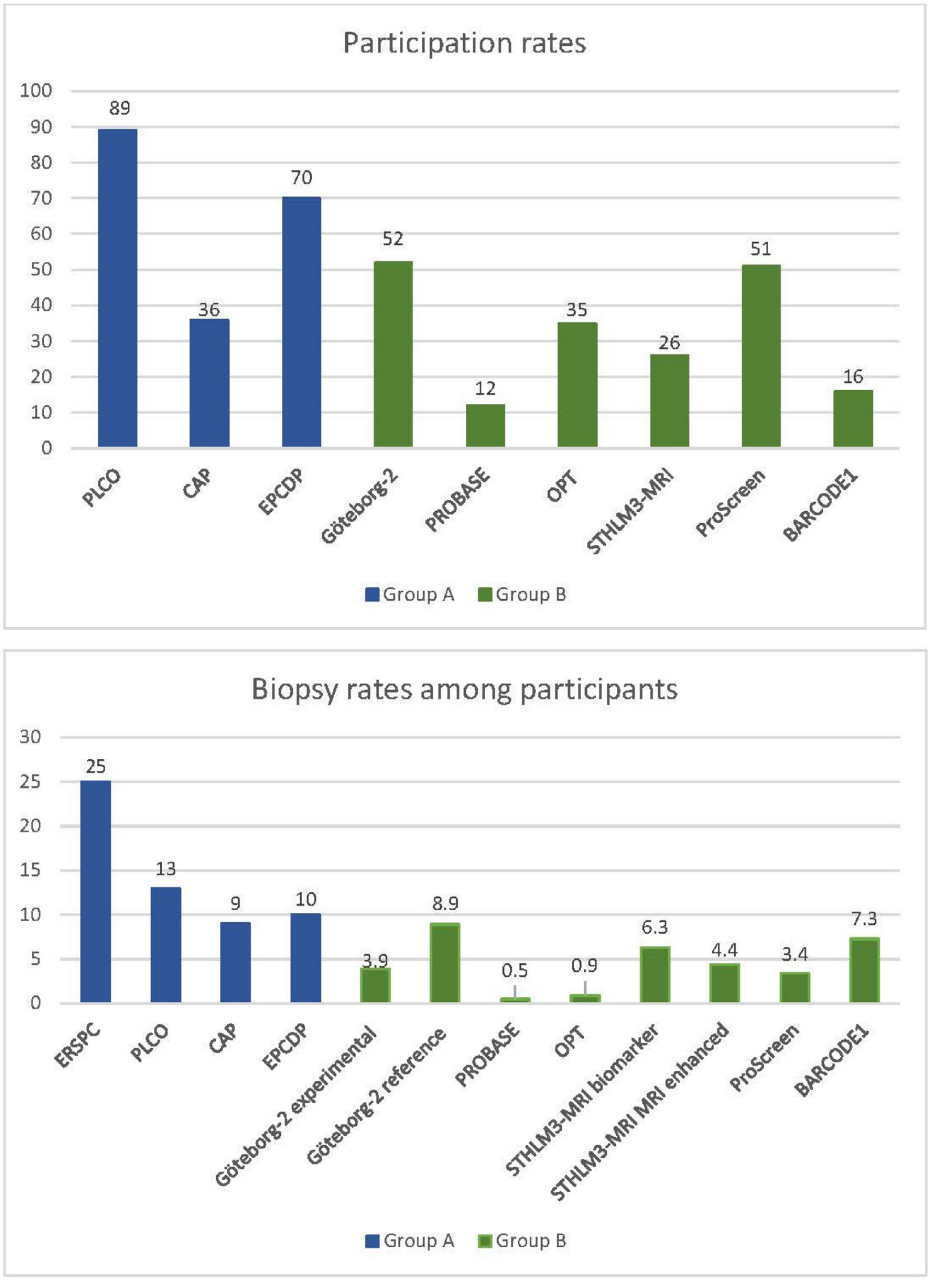
Participation rates and proportions of biopsies performed in participants of groups A and B. ERSPC = European Randomised Study of Screening for Prostate Cancer; PLCO = Prostate, Lung, Colorectal and Ovarian Cancer Screening Trial; CAP = Cluster randomised trial of PSA testing for Prostate cancer; EPCDP = Early Prostate Cancer Detection Programme; PROBASE = Prostate Cancer Early Detection Study Based on a “Baseline” PSA value in Young Men; OPT = Organised Prostate cancer Testing; PSA = prostate-specific antigen, MRI = magnetic resonance imaging.

Participation rates for each study are displayed in Fig. 2, ranging from 12% to 89%. For randomized controlled trials, the participation rates reflect those of the intervention arm, ranging from 36% to 89%. Within the ERSPC study, participation rates varied between 74% and 100% across different study centers. For the more contemporary population-based screening trials (not randomized), participation rates ranged from 12% to 70%.

##### 3.3.1.1 Interpretation

When comparing participation rates, it is important to consider the various factors affecting these. Participation rates were notably higher in older trials than in contemporary trials (group B), none of which exceeded 52%, with most reporting participation rates of <50% (Fig. 2).

A possible explanation for the higher participation rates in older trials such as ERSPC and PLCO is the low rate of opportunistic PSA screening in early years in combination with increased social interest for a novel PSA screening program.

Contemporary data show a completely different picture [30], which may lead to reduced interest in participating in screening programs nowadays.

Variations in study design may also explain the wide range of participation rates. Studies can have either an efficacy design or a population-based effectiveness design [31]. In efficacy studies, participants are randomized after they choose to participate, often resulting in higher participation rates. In contrast, effectiveness studies randomize individuals before inviting them, which reflects real-world conditions better but leads to lower participation rates. Efficacy designs offer a controlled environment with high internal validity, as results are less likely to be influenced by external factors, but have limited generalizability due to a selection bias [32]. This difference in participation rates is reflected by the range within the ERSPC study (range 74–100%), as described by Hugosson et al [33]. They found that countries with an effectiveness design (the Netherlands, Belgium, Switzerland, and Spain) generally have higher participation rates (95%, 91%, 97%, and 100%, respectively) than countries with an effectiveness design (Finland, Sweden, and Italy with 74%, 76%, and 79% participation rates, respectively) [14,33].

Next to different designs, age at the time of participation may also play a role. In this review, it was found that participation rates appeared to be lower in relatively younger cohorts than in older cohorts (PROBASE: age 45–50 yr, participation 12%; OPT: age 50 yr, participation 35%). This is in line with a pilot study from Alterbeck et al [34] researching participation rates among different age groups, which noted an increasing participation rate in the older age groups. Heijnsdijk et al [35] conducted a simulation study, concluding that PCa screening could be cost effective with two or three rounds of PSA tests between the ages of 55 and 59 yr. This model assumed a participation rate of 80%. However, our review suggests that this assumption may be questionable, as the participation rates we observed are significantly lower than 80%, raising concerns about the feasibility of initiating screening at age 55 yr or even 50 yr. Creating awareness among men that screening is not something reserved for the elderly is important.

The PROBASE study identified several reasons for their relatively low participation rate (12%) [36]. The most common reason the nonparticipants cited was a lack of interest (69%). This seems to be a population-wide problem, because participation rates in other screening programs in Germany are also low. In addition, the German Statutory Early Detection Program recommends an annual DRE starting at age 45 yr, which can be reimbursed by the German health insurance. However, this is not the case for PSA testing and therefore potentially contributing to a lower participation rate in this study. Similarly, the PROSHADE study, in which Spanish men over 40 yr were surveyed, found that despite good awareness of PCa, knowledge about the PSA test is limited, indicating a need for better education on PCa screening [37].

#### 3.3.2 PSA positives

The proportions of PSA positives were found to be in the range of 0.8–29%. The threshold for further diagnostics was set at PSA ≥3 ng/ml for all studies, except for the PLCO and BARCODE1 studies, which used a threshold of PSA ≥4 ng/ml and PRS ≥90th percentile, respectively.

##### 3.3.2.1 Interpretation

Several factors might have an impact on the number of PSA positives. First of all, there is a strong correlation between age and PSA [38]. This is reflected in the low PSA-positive rates observed in the trials that included young men, that is, the PROBASE (age 45 yr) and OPT (age 50 yr) trials, reporting 0.8% and 2.9% rates, respectively.

Another explanation is the use of a repeat PSA test, as is done in the PROBASE trial. If the initial PSA test is elevated, a second conclusive test is performed 2 wk afterward, leading to fewer PSA positives than in other trials. A recent study by Davik et al [39] found that a repeat PSA test provides valuable predictive information for men undergoing MRI and prostate biopsy. Men with a lower PSA level on the repeat test have a significantly lower risk of detecting csPCa and PCa. The European Association of Urology (EAU) guidelines mention that a repeat PSA test is recommended prior to further diagnostics for asymptomatic men with a PSA level of 3–10 ng/ml and normal DRE [40].

Moreover, the geographic location of the studies and the socioeconomic status of the participants partially influence the number of PSA positives. Men from disadvantaged areas tend to experience a higher burden of PCa, possibly due to factors such as limited access to health care, delayed diagnosis, and a higher prevalence of risk factors. This may contribute to variations in PSA positivity rates observed across different studies [41]. These demographic factors should be taken into account when comparing this PI.

#### 3.3.3 Proportions of MRI scans performed and MRI compliance

The proportion of MRI scans performed among participants was found to be in the range of 0.6–11%, with a rate of compliance to MRI ranging from 79% to 97%. MRI was not performed within group A (ERSPC, PLCO, CAP, and EPCDP).

##### 3.3.3.1 Interpretation

As already mentioned, new diagnostic steps have been integrated into the PCa screening pathway over the past decades. The EAU updated its guidelines in 2019 to recommend prebiopsy MRI for biopsy-naïve men [2]. This was partly driven by the results of the PRECISION trial, in which it was found that the usage of prebiopsy mpMRI and MRI-targeted biopsy led to an increase in the detection of csPCa compared with standard biopsy and a decrease in the detection of insignificant PCa [42].

Within group B, the percentage of participating men who eventually underwent MRI was found to be in the range of 0.6–11%. Several components contribute to this diversity. The most logical explanation for the observed differences is the variation in the number of multivariable algorithm positives. For example, fewer MRI scans were conducted in OPT and PROBASE (2.7% and 0.6%, respectively), which also reported fewer PSA positives (2.9% and 0.8%, respectively) than other studies. As mentioned in the previous section, the lower mean age of participants in these trials (OPT and PROBASE) probably reduces the likelihood of triggering further diagnostic steps. Another example is the difference in compliance. In the PROBASE study, for instance, the compliance rate was 79%, which is lower than in other studies and consequently resulted in fewer MRI scans being performed. However, the overall compliance to MRI is remarkably high (79–97%), which is encouraging and may support the successful implementation of future screening programs. The advantages of MRI include reducing unnecessary biopsies and minimizing the overdiagnosis of low-grade disease in men with negative MRI results. Additionally, MRI enables the targeted biopsy of specific lesions that might be missed with a systematic biopsy [43]. However, prebiopsy MRI and a targeted biopsy lead to upgrading of tumors compared with a systematic biopsy [44]. Taking multiple targeted cores from a lesion to which the highest Gleason score applies automatically results in a higher grade than systematic biopsy. This so-called grade shift, in turn, requires more treatment.

#### 3.3.4 Proportions of biopsies performed and biopsy compliance

The rate of compliance to biopsy ranged from 36% to 99%. The biopsy rates among participants are displayed in Fig. 2, ranging from 0.5% to 25%.

##### 3.3.4.1 Interpretation

The wide variation in terms of biopsy can be caused by a combination of factors. To begin with, it is evident that more biopsies were performed in group A than in group B (Fig. 2). This can easily be attributed to the differences in the diagnostic algorithms of the protocols. In group A, all participants with a positive PSA result were immediately referred for a biopsy, while in group B, MRI was used as an intermediate step before proceeding to a biopsy. But even within group B, there were variations. For example, in the OPT study, MRI was followed by an additional risk stratification step, resulting in only 0.9% of participants undergoing a biopsy. In contrast, in the STHLM3-MRI biomarker group, a biopsy was performed in every participant with a positive PSA result, and in the Göteborg-2 reference group, a biopsy was performed in all PSA-positive participants regardless of MRI findings.

Second, as with MRI, biopsy rates are also affected by participants' compliance. This partly explains the notable difference in biopsy rates between the ERSPC and PLCO studies (25% and 13%, respectively), as adherence was significantly higher in the ERSPC study (86% and 45%, respectively). In the PLCO study, the decision to perform biopsies was left to regional health care providers, while the ERSPC followed strict protocols that ensured more consistent biopsy practices [45].

#### 3.3.5 PCa detection rates and csPCa rates

##### 3.3.5.1 Detection of PCa

The positive predictive value (PPV) of PCa for biopsy ranged from 33% to 82%. Additional information was available on interval cancers and cancers among nonattenders in ERSPC, CAP, and ProScreen, and on cancers detected outside the screening program in EPCDP. The proportions of non–screen-detected cancers among total detected PCa cases were 41% for ERSPC, 45% for CAP, 26% for ProScreen, and 40% for EPCDP.

It was not possible to calculate the PPV for PLCO and CAP.

##### 3.3.5.2 Detection of csPCa

**Fig. 3:**
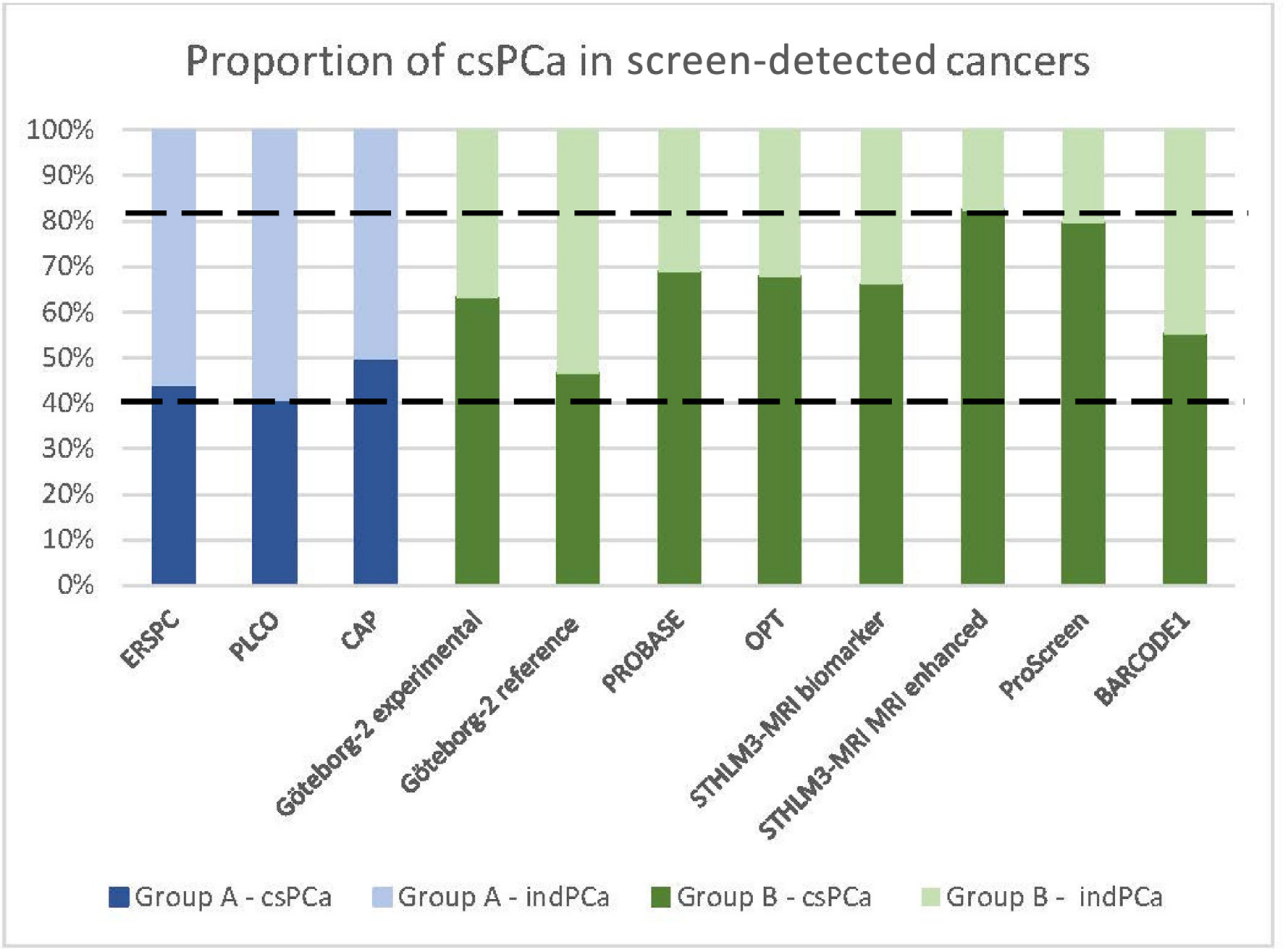
Proportion of detected indolent PCa (indPCa) and clinically significant prostate cancer (csPCa) cases for the different studies. ERSPC = European Randomised Study of Screening for Prostate Cancer; PLCO = Prostate, Lung, Colorectal and Ovarian Cancer Screening Trial; CAP = Cluster randomised trial of PSA testing for Prostate cancer; EPCDP = Early Prostate Cancer Detection Programme; PROBASE = Prostate Cancer Early Detection Study Based on a “Baseline” PSA value in Young Men; OPT = Organised Prostate cancer Testing; PSA = prostate-specific antigen, MRI = magnetic resonance imaging.

Fig. 3 displays the proportion of screen-detected csPCa (defined as Gleason ≥7) for groups A and B, ranging from 41% to 82%. This information was not available for EPCDP.

##### 3.3.5.3 Interpretation

The cancer detection rates of biopsies along with, if known, the number and percentage of detection of grade group (GG) ≥2 PCa of each study were described. For the experimental group of the Göteborg-2 study, targeted biopsies were performed. This yielded a biopsy cancer detection rate of 66%, of which 63% were considered clinically significant, compared with 46% and 47%, respectively, in the reference group where only systematic biopsies were performed. Similar trends were observed in the biomarker group of the STHLM3-MRI study, in which systematic biopsies were conducted for all high-risk participants. This arm showed a lower rate of biopsy cancer detection (55%), of which 66% was considered clinically significant, compared with the MRI-enhanced group, where only targeted biopsies were performed for MRI-positive results, showing higher rates for both overall and csPCa detection rates (69% and 82%, respectively). This is in line with the results from a systematic review and meta-analysis performed by Xie et al [46] researching targeted versus systematic biopsies. In total, 26 studies were analyzed, and it was found that a targeted biopsy detects more csPCa cases than a systematic biopsy.

## 4 Discussion

We provided an overview of the different study designs and PIs for the most well-known PCa screening trials of the past decades, to serve as a reference for ongoing screening initiatives. It is widely acknowledged that PCa screening represents a complex harm-to-benefit ratio. Despite the evidence that screening reduces PCa-specific mortality [47], there is reluctance to implement national screening programs due to the presence of anxiety, unnecessary biopsies, overdiagnosis, and overtreatment.

Although the detection of GG1 PCa is relevant in the context of overdiagnosis, we did not include this as a PI in our analysis, as current PCa screening studies mostly focus on the detection of csPCa. As such, csPCa detection is more commonly used as a benchmark for evaluating screening effectiveness across studies and aligns with the PIs prioritized in recent initiatives, such as PRAISE-U.

In this study, we found that many factors impact the different screening PIs. One of the most important contributing factors to consider is age, when comparing PIs from PCa screening trials. It was found in this review that there is an impact of age in every diagnostic step. Older men are more likely to participate in PCa screening and higher PSA levels in this group can lead to more follow-up procedures such as MRI and biopsy.

Understanding the characteristics of a screening pilot/trial is necessary to evaluate the delivered PIs properly. Additionally, it is important to consider evolving guidelines around PCa screening in both Europe and America over the past decades [2].

This study knows several limitations. First, variations in follow-up across different trials could potentially affect cancer detection rates. Moreover, heterogeneity between studies complicates direct comparisons and undermines the certainty of the conclusions drawn. In particular, not all PIs were available uniformly across studies. This highlights the necessity of clear guidelines on PCa screening PIs. Lastly, there may be a publication bias, with included studies potentially publishing data which may lead to an overall positive view of PCa screening. However, large population-based PCa screening studies from the past published both positive and negative results (CAP [*N* = 415 357], ERSPC [*N* = 162 241], and PLCO [*N* = 76 693]).

As mentioned previously, implementation of population-based screening for PCa remains limited for several reasons. According to the Wilson and Jungner [5] criteria, PCa meets several key conditions for a screening program: it represents a significant and growing public health burden, it can be detected at an early and more treatable stage, and it has a measurable impact on morbidity and mortality. However, the PSA test, while sensitive, has low specificity, leading to a high rate of false positives and overdiagnosis. Current screening algorithms still result in the detection of indolent cancers, and as a result, population-wide screening is not yet considered cost effective.

In the context of shifting screening protocols and recommendations, the PRAISE-U project is initiated to provide valuable insights into the implementation of PCa screening programs for the future. A comparison with group B from this study can be made for describing the expected results, excluding results from PROBASE because of the difference in age of the participants (PROBASE age 45 yr vs PRAISE-U 50–69 yr). Hence, based on our results, the participation rate is expected to be between 10% and 50%. Pilots will use different methods of invitation (text message, e-mail, and physical letter), and nonparticipants will be surveyed to investigate an optimal screening strategy. The percentage of PSA positives is expected to be <15%. Two risk stratification steps will be incorporated in the screening process. The first risk stratification step will take place after the PSA measurement in order to reduce referrals to MRI caused by benign elevation of serum PSA. The second risk stratification step will be done after MRI. By doing so, the expectation is to reduce the number of prostate biopsies, that is, reducing invasive testing to reduce harm and possible overdiagnosis. The rate of prostate biopsies in all men participating in a population-based program is expected to be well below 10%, with an expected detection rate of csPCa well above 50–60%.

## 5 Conclusions

The presented PIs provide a framework for comparing current and future PCa screening data with those of past trials. Despite the fact that defining PIs is considered a valid method to make comparisons, it must be noted that our work also made it clear that numerous factors have an effect on PIs in screening trials. It remains crucial to consider the different populations, methods for invitation, screening protocols, and screening techniques (such as the use of MRI and risk stratification) and their potential impact on PIs.

  ***Author contributions:*** Meike J. van Harten had full access to all the data in the study and takes responsibility for the integrity of the data and the accuracy of the data analysis.

  *Study concept and design*: van Harten.

*Acquisition of data*: van Harten.

*Analysis and interpretation of data*: van Harten, van den Bergh, Roobol, Willemse.

*Drafting of the manuscript*: van Harten.

*Critical revision of the manuscript for important intellectual content*: van den Bergh, Roobol, Willemse.

*Statistical analysis*: None.

*Obtaining funding*: None.

*Administrative, technical, or material support*: None.

*Supervision*: van den Bergh, Roobol, Willemse.

*Other*: None.

  ***Financial disclosures:*** Meike J. van Harten certifies that all conflicts of interest, including specific financial interests and relationships and affiliations relevant to the subject matter or materials discussed in the manuscript (eg, employment/affiliation, grants or funding, consultancies, honoraria, stock ownership or options, expert testimony, royalties, or patents filed, received, or pending), are the following: None.

  ***Funding/Support and role of the sponsor:*** None.
